# Cost-effectiveness and budget impact of adding tranexamic acid for management of post-partum hemorrhage in the Indian public health system

**DOI:** 10.1186/s12884-022-05308-4

**Published:** 2023-01-06

**Authors:** Beena Nitin Joshi, Siddesh Sitaram Shetty, Kusum Venkobrao Moray, Himanshu Chaurasia, Oshima Sachin

**Affiliations:** 1 Department of Operational Research, Indian Council of Medical Research - National Institute for Research in Reproductive and Child Health, Mumbai, Maharashtra India; 2 Department of Population Health Sciences, King’s College London, Ex-Regional Resource Hub for Health Technology Assessment, Indian Council of Medical Research - National Institute for Research in Reproductive and Child Health, Mumbai, Maharashtra India; 3 Ashwini Rural Medical College Hospital and Research Institute, Kumbhari, Solapur, Ex - Regional Resource Hub for Health Technology Assessment, Indian Council of Medical Research - National Institute for Research in Reproductive and Child Health, Mumbai, Maharashtra India; 4 Regional Resource Hub for Health Technology Assessment, Indian Council of Medical Research - National Institute for Research in Reproductive and Child Health, Mumbai, Maharashtra India; 5 Department of Health Research, Ministry of Health and Family Welfare, New Delhi, India

**Keywords:** Postpartum hemorrhage, Tranexamic acid, Cost-effectiveness, Maternal mortality, Primary PPH management, IV TXA

## Abstract

**Background:**

Postpartum hemorrhage (PPH) is the global leading cause of maternal mortality, affecting nearly 3 to 6 percent of all women giving birth in India. The World Health Organization (WHO) has updated its guidelines to recommend the early use of intravenous (IV) tranexamic acid (TXA) in addition to standard care for all diagnosed PPH cases. This study aimed to assess the cost-effectiveness of introducing TXA for PPH management in the Indian public health system.

**Methods:**

A decision analytic model was built using a decision tree to determine the cost-effectiveness of administering IV TXA to women experiencing PPH within 3 h of birth to existing management with uterotonics and supportive care. Using a disaggregated societal perspective, the costs and consequences for a hypothetical cohort of women experiencing PPH in public health facilities was estimated. The model was populated using probabilities, clinical parameters, and utilities from published literature, while cost parameters were largely derived from a primary economic costing study. The primary outcome of interest was the incremental cost-utility ratio (ICUR). Associated clinical events and net benefits were estimated. One-way and probabilistic sensitivity analysis (PSA) was undertaken. The budget impact was estimated for a national-level introduction.

**Results:**

For an estimated annual cohort of 510,915 PPH cases in India, the addition of IV TXA would result in a per-patient disaggregated societal cost of INR 6607 (USD 95.15) with a discounted gain of 20.25 QALYs, as compared to a cost of INR 6486 (USD 93.41) with a discounted gain of 20.17 QALYs with standard care PPH management. At an ICUR value of INR 1470 per QALY gained (USD 21), the addition of IV TXA is cost-effective in Indian public health settings. The intervention is likely to prevent 389 maternal deaths, 177 surgeries, and 128 ICU admissions per 100,000 PPH cases. The findings are robust under uncertainty, with 94.5% of PSA simulations remaining cost-effective. A cumulative increase of 2.3% financial allocation for PPH management over five years will be incurred for TXA introduction.

**Conclusions:**

Addition of tranexamic acid for primary PPH management, as recommended by WHO, is cost-effective in Indian public health settings. Policy guidelines, training manuals, and facility checklists should be updated to reflect this recommendation.

**Supplementary Information:**

The online version contains supplementary material available at 10.1186/s12884-022-05308-4.

## Background

Target 3.1 of the Sustainable Development Goals (SDG) is to reduce the global maternal mortality ratio (MMR) to less than 70 per 100,000 live births by the year 2030 [1]. Global efforts have resulted in an MMR reduction over the years; however, it still remains high in certain parts of the world [2, 3]. Sub-Saharan Africa and South Asia accounted for approximately 86% of global maternal deaths in 2017 [2]. Maternal hemorrhage, hypertensive disorders, and sepsis are the worldwide leading causes of maternal morbidity and mortality and are largely preventable or treatable [4, 5]. Improving maternal health saves lives, is an indicator of health system well-being, has larger socio-economic benefits, and potentially rewards the health system with a considerable return on investment [6, 7]. However, health systems only have a finite pool of resources available, and prioritization based on efficiency and effectiveness is highly warranted.

India has reported an impressive decline in MMR, with the national Sample Registration System (SRS) reporting a value of 103 per 100,000 live births in SRS 2017–19 [8]. The Indian government has reported being on track in achieving national and SDG targets and has highlighted the benefits of strategically investing in a range of interventions such as the Labor Room Quality Improvement Initiative (LaQshya), *Janani Suraksha Yojana* (JSY), etc. [9]. Despite ongoing progress, India accounted for the second highest number of maternal deaths, i.e., 12 percent of the global numbers in 2017 [10]. In line with global trend, hemorrhage remains the leading cause of maternal deaths in India [11–13]. Post-partum hemorrhage (PPH) is defined as blood loss of 500 ml or more within 24 h after birth and is the primary leading cause of maternal mortalities in India and across the world [14, 15]. PPH affects nearly 3–6 percent of all deliveries [15, 16]. Prevention and timely provision of treatment for post-partum hemorrhage by a well-equipped health system can be vital to improving maternal health outcomes and enhancing the overall childbirth experience for women availing healthcare services.

Management of PPH in India and across the globe is largely based on principles of maintaining a continuum of care beginning with preventive and first-line treatment using uterotonics, moving to temporizing measures (e.g., uterine massage, uterine balloon tamponade, etc.), and ending with emergency surgical care (e.g., devascularization group of surgeries, hysterectomy, etc.) if required [17–20]. Ensuring concurrent resuscitation, monitoring, diagnosis and cause-specific treatment measures using a multi-disciplinary approach is essential. In 2017, based largely on findings of the World Maternal Antifibrinolytic (WOMAN) Trial, the World Health Organization (WHO) updated its guidance to recommend early use of intravenous tranexamic acid (TXA) (within 3 h of birth) as part of a standard treatment package for all clinically diagnosed PPH cases [21]. This recommendation is also echoed by PPH management guidance offered by the Royal College of Obstetricians and Gynecologists (RCOG) in 2016, and the International Federation of Gynecology and Obstetrics (FIGO) in 2022 [20, 22].

The existing Indian guidelines on PPH management framed in 2015 recommend using tranexamic acid if oxytocin and other uterotonics fail to stop bleeding or if bleeding is partly due to trauma [19]. The updated WHO recommendation has not yet been reflected in Indian guidelines. Similarly, public initiatives such as the DAKSHATA program that focuses on improving quality of maternal and newborn care do not yet have tranexamic acid drug mentioned in the medicine tray or in the PPH management kit expected to be available in every labor room for standardization of services [23, 24]. For lack of guidance, a policy question regarding use of intravenous tranexamic acid in the management of all PPH types in the Indian public health system was proposed by the state public health department, Government of Maharashtra. An economic evaluation with budget impact implication for such an introduction was thus undertaken to answer this policy question [25]. A systematic review undertaken in 2021 assessing cost-effectiveness evidence for tranexamic acid in PPH management identified only four studies (two abstracts) from three countries (United States, Nigeria, & Pakistan) to recommended more studies to be undertaken in different populations and settings to inform policy decisions [26]. Since this systematic review, to our knowledge, another cost-effectiveness analysis from the United States has been reported [27]. This present study aims to bridge the existing cost-effectiveness and budget impact evidence gap specifically for a developing country context like India having high PPH prevalence.

## Methods

A decision tree type of decision analytic model was developed to determine the cost-effectiveness of early addition (within three hours of delivery) of intravenous tranexamic acid to standard care treatment with uterotonics and supportive care for all PPH cases. The decision model was conceptualized using principles of the Indian HTAIn reference case [28]. A disaggregated societal perspective, i.e., health system, along with an immediate patient perspective was chosen for the model. The population included a hypothetical cohort of women in the reproductive age group of 15–49 years with median a age of 21 years at first childbirth experiencing PPH after delivery at any Indian public health facility [29]. The intervention assessed was the addition of intravenous (IV) TXA to standard care treatment. IV TXA (one gram in 10 ml or 100 mg/ml at one ml per minute) administered over 10 min within three hours of birth with the second dose of one gram IV if bleeding continues after 30 min or if it restarts within 24 h of completion of the first dose for all primary PPH cases as adopted by the WOMAN trial study was considered. The comparator, i.e., standard care treatment alone, includes first-line treatment with intramuscular or intravenous oxytocin, followed by intravenous methergine or sublingual misoprostol if oxytocin is unavailable. Ongoing fluid resuscitation and supportive interventions are provided before moving to surgical interventions. Indian guidelines recommend management depending on the type of PPH experienced and the level of healthcare facility accessed by women, as shown in the subsequent structure of the decision model. The primary outcome was to assess the incremental cost-utility ratio (ICUR), i.e., the incremental cost per incremental Quality Adjusted Life Years (QALY) gained with the addition of TXA intervention. Secondary outcomes included an assessment of cost-effectiveness in the net benefit framework, number of maternal deaths, surgeries, and ICU admissions associated respectively with intervention and standard care. A lifetime horizon was considered appropriate for the model to account for mortality and associated long-term consequences, especially due to emergency lifesaving hysterectomy that is relevant to the Indian context.

### Model structure

**Fig. 1:**
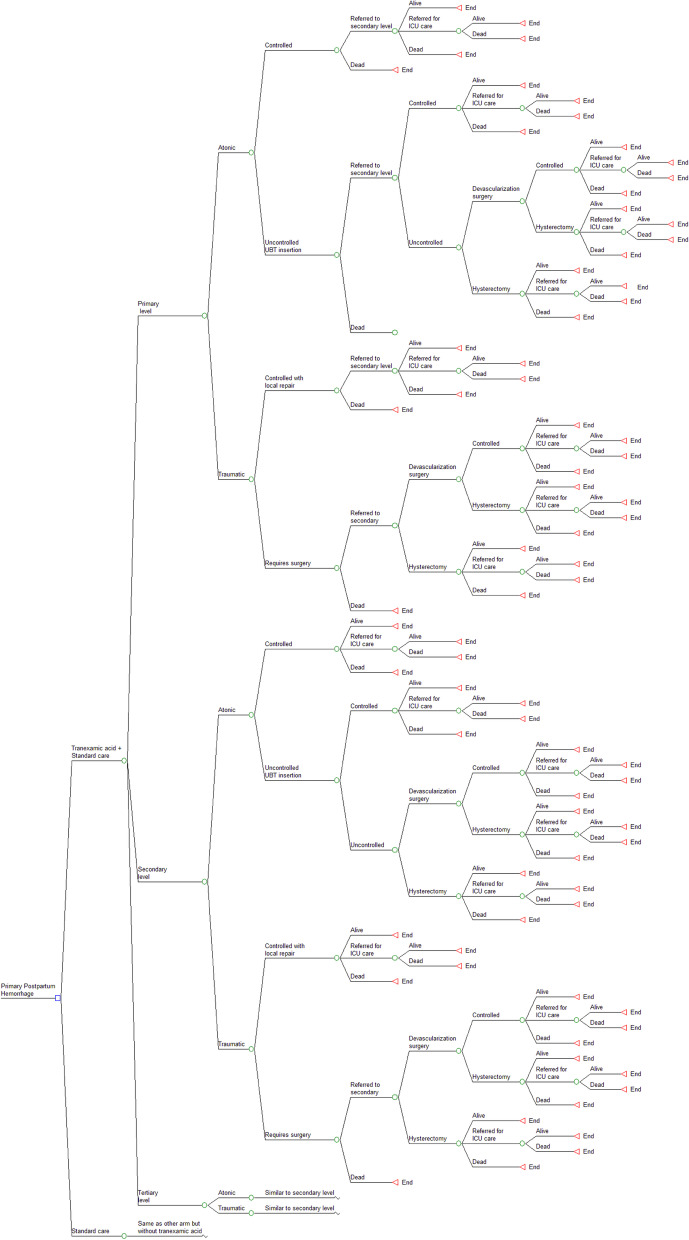
Decision tree diagram comparing intravenous Tranexamic acid (TXA) addition to standard care management for PPH

The model was developed using Microsoft Excel 2016 with Microsoft Visual Basic for Application 7.1. The model, shown in Fig. 1 begins with an annual cohort of women experiencing PPH during childbirth in Indian public health facilities. At the decision node, the tree splits between standard care or the addition of TXA to standard care as the intervention arm. The same pathway is followed by both decision arms except for the addition of TXA drug to medical management for all cases in the intervention pathway. The decision tree branches out based on the level of healthcare facility accessed by women and depending on the type of PPH experienced. For atonic PPH, the model considers the failure of medical management to control bleeding to be followed by the use of uterine balloon tamponade (UBT) intervention as a conservative measure recommended by Indian and WHO guidelines [17, 30]. Depending further on the control of bleeding, either devascularization, i.e., uterine salvaging procedure like B-Lynch suturing, arterial ligation, or a lifesaving hysterectomy, directly or subsequently after conservative procedures, may be considered for uncontrolled cases. For traumatic PPH, it is expected that the source of bleeding will be explored and repaired either locally or using surgical procedures as stated before. Following the clinical course, the pathway terminates with ‘alive’ or ‘dead’ terminal nodes. The model assumes that those availing services at the primary level, i.e., primary healthcare centre (PHC) will have facilities in the form of a medical officer or a skilled birth attendant to initially stabilize and refer patients to secondary care. Secondary level, i.e., community health centres and sub-district hospitals, are additionally equipped with obstetric specialists, operating facilities, and blood transfusion services to manage PPH. Those requiring intensive care unit (ICU) admission from primary and secondary levels are expected to be referred to district hospitals or medical colleges, which constitute the tertiary level of care. The model assumes that all health facilities are well-equipped with trained staff and that all PPH cases requiring specific interventions will receive them in a timely manner.

### Probabilities

An initial review of literature to determine the clinical effectiveness of TXA identified existing published reviews. A review of these reviews using predefined PICO criteria identified a Cochrane review, three systematic reviews, and five targeted reviews. An RCT of 144 women by Ducloy Bouthers et al. and the WOMAN trial were the most commonly reported studies identified across reviews [31, 32]. The WOMAN trial, as the single largest multi-country, multi-centric, placebo-controlled study of 20,060 women from across 193 hospitals in low-, middle- and high-income countries largely based on which the WHO has framed guidance and dosage recommendations was chosen to populate clinical parameters related to the effectiveness of TXA in the model. The relative risk of controlling PPH bleeding with TXA as compared to standard care without any further interventions as a measure of clinical effectiveness was estimated using findings reported on conservative or surgical interventions across the trial arms. The relative risk of death due to bleeding and all cause-mortality between intervention and standard care arms were similarly obtained from the study. A detailed explanation of this calculation is given as supplementary material (see Additional file 1). Other input parameters, such as the proportion of cases requiring UBT intervention at specific healthcare levels, and the proportion of cases undergoing specific surgical interventions were obtained from literature relevant to the Indian context [33, 34]. The clinical effectiveness of UBT intervention and ICU admission rates were obtained from available published studies [35, 36]. Table 1 lists clinical input parameters along with their upper and lower limits.

**Table 1:** Model input parameters with upper and lower limits for sensitivity analysis

Input parameter	Base-case value	Upper limit	Lower limit	Source
Age start	21 years ^a^	16.8	25.2	[29]
The proportion of PPH cases that are atonic	0.80 ^a^	0.64	0.96	[37]
The relative risk of further interventions with TXA	0.96 ^a^	0.76	1.15	Calculated from [32]
The relative risk of further interventions with SOC	1.05	0.84	1.26	Calculated from [32]
Risk of further intervention events with TXA	0.22	0.18	0.27	Calculated from [32]
Risk of further intervention events with SOC	0.23 ^a^	0.19	0.28	Calculated from [32]
UBT insertion for atonic PPH at primary	0.19 ^a^	0.15	0.22	[29]
UBT insertion for atonic PPH at secondary	0.33 ^a^	0.26	0.40	[29]
UBT insertion for atonic PPH at tertiary	0.48 ^a^	0.39	0.58	[29]
Clinical effectiveness of condom-UBT	0.92 ^a^	0.74	0.98	[35]
The relative risk for death due to bleeding with TXA	0.69	0.52	0.91	Calculated from [32]
Risk of death due to bleeding with standard care (SOC)	0.02 ^a^	0.01	0.02	Calculated from [32]
Direct hysterectomy for uncontrolled atonic PPH after UBT intervention	0.15 ^a^	0.12	0.18	[34]
Hysterectomy after devascularization	0.22 ^a^	0.18	0.26	[34]
The relative risk for all-cause mortality with TXA	0.88	0.74	1.05	Calculated from [32]
Risk of all-cause mortality rate with standard care	0.03 ^a^	0.02	0.03	Calculated from [32]
Risk of ICU admission for UBT uncontrolled cases	0.77 ^a^	0.62	0.92	[36]
Risk of ICU admission for UBT-controlled cases	0.03 ^a^	0.02	0.03	[36]
Traumatic PPH controlled with suturing and local measures	0.45 ^a^	0.36	0.55	[34]
Cost of medical management with TXA at the primary level	451	353	560	From the Primary costing study
Cost of medical management with TXA at the secondary level	1685	1405	1945	
Cost of medical management with TXA at tertiary level	2812	2433	3176	
Cost of medical management with SOC at the primary level	241	139	353	
Cost of medical management with SOC at the secondary level	1475	1207	1740	
Cost of medical management with SOC at tertiary	2601	2171	3053	
Cost of UBT insertion for atonic PPH at the primary level	281	254	308	
Cost of UBT insertion for atonic PPH at the secondary level	531	446	618	
Cost of UBT insertion for atonic PPH at tertiary level	1303	1170	1433	
Cost of devascularisation surgery at the secondary level	4991	3986	5997	
Cost of devascularisation surgery at tertiary level	8271	6483	10,109	
Cost of hysterectomy at the secondary level	7535	6150	9016	
Cost of hysterectomy at tertiary level	11,462	9529	13,343	
Cost of indoor patient (IPD) admission for PPH at the secondary level	2230	1290	3310	
Cost of IPD admission for PPH at tertiary level	3273	2387	4236	
Cost of ICU admission for PPH at the tertiary level	9901	4759	15,731	
Cost of local management for traumatic at primary	264	177	351	
Cost of local management for traumatic at secondary	387	302	470	
Cost of local management for traumatic at tertiary	689	532	851	
Cost of patient referral	1096 ^a^	876	1315	[38]
Out-of-pocket expenditure for childbirth	3015 ^a^	2412	3618	[39]
Number of TXA doses	1.29	1.28	1.30	[40]
Utility at death	0.00	0	0	
Utility at discharge (medical management)	0.930	0.910	0.940	[40]
Utility for medical plus conservative measures	0.895	0.892	0.897	[40]
Utility short-term devascularization (42 days)	0.565 ^a^	0.300	0.870	[41]
Utility devascularization long term (Beyond 42 days)	0.909	0.500	0.960	[41]
Utility hysterectomy short-term (42 days)	0.560 ^a^	0.448	0.672	[42]
Utility hysterectomy long-term (beyond 42)	0.880 ^a^	0.704	1.000	[42]
Utility ICU admission (1.5 days)	0.490^a^	0.392	0.588	[42]
Discount rate	0.030	0.000	0.050	[43]

^a^The upper and lower limit for these parameters are calculated by assuming a 20 percent variation on both sides. For remaining parameters, limits are obtained either from primary sources or by calculating confidence interval limits

### Costs

Cost and resource utilization data were collected as part of a primary bottom-up micro-costing exercise for estimation of health system cost related to PPH management. Data from one PHC, one sub-district hospital, one district hospital, and one government medical college from the state of Maharashtra, India, representing all three levels of care was used to calculate the cost for various components of PPH management with TXA intervention [35]. Health system unit cost packages included the cost of medical management with and without TXA, UBT procedure cost across healthcare levels, local management of traumatic PPH, surgical interventions, and indoor patient and ICU admissions. Cost data initially collected for the year 2017–18 was inflated to year 2019–20 using standardized methods [44]. The cost of tranexamic acid drug was calculated by assuming utilization of 1.29 mean doses per patient as reported by the WOMAN trial and attaching a TXA drug price of INR 132.9 (USD 1.91) for 1gm/10 ml obtained from the 2016 National Pharmaceutical Pricing Authority (NPPA) compendium [40, 45]. The economic cost of providing intervention was assumed to be covered by the estimated unit cost of medical management in addition to TXA drug price. The non-specific package cost estimated for obstetric indoor patient and ICU admissions respectively were apportioned for PPH management using an average length of stay of 2.9 and 1.5 days as reported in literature [36, 46, 47]. Due to lack of primary data, referral cost was obtained from an Indian-published study [38]. To account for disaggregated societal costs, a nationally representative out-of-pocket expenditure cost incurred by families on account of childbirth was used [39]. Table 1 enlists unit cost parameters used in the model.

### Consequences

QALY estimation was the primary health outcome of interest. To calculate QALY for each terminal node, utility weights for associated events such as ICU admission of 1.5 days, short-term (42 days post-partum) and long-term utility of undergoing devascularization or hysterectomy surgery, utility at discharge after PPH or utility associated with death as applicable to each terminal node were applied to the remaining period of an overall life-expectancy of 70.7 years for Indian females to estimate QALYs associated with each arm of the decision tree [48]. Utility scores as reported in Table 1 were obtained from available published sources.

### Analysis

ICUR value which is incremental cost per QALY gained with the addition of TXA to standard care was estimated by comparing overall costs and outcomes associated with two decision tree pathways. All unit costs used in the model were adjusted to the year 2019–20. Using an annual cohort of births occurring in Indian public health facilities in the year 2019–20 and the probability of various clinical events in the decision tree, secondary clinical outcomes such as the number of PPH-associated surgeries, ICU admissions, and maternal deaths were calculated. As recommended by Indian HTAIn reference case, a discount rate of 3 percent was used. Costs incurred on account of the PPH event are expected to be a one-off event and hence were kept undiscounted whereas outcomes over a lifetime were discounted for the primary endpoint of analysis. However, both discounted and undiscounted results from the health system and disaggregated societal perspective as recommended in India are reported. The cost-effectiveness threshold value was set at Indian National Rupees (INR) 145,742 i.e. United States dollars (USD) 2099 reflecting the Indian gross domestic product (GDP) per capita for the year 2019–20 [49]. For the conversion of costs in USD, a 2019 exchange rate of 1 USD = 69.43 INR was used [50].

Both one-way (OWSA) and probabilistic sensitivity analysis (PSA) was undertaken to address model uncertainties. Upper and lower limits for clinical and utility score parameters if available from original sources were used or else a 20 percent variation on either side was assumed. For each unit cost, a 95% confidence interval (CI) limit was derived by running 1000 Monte Carlo simulations for each unit cost subcomponent with assigned distributions. These limits were then used in varying unit costs in the sensitivity analysis. A beta distribution was assigned for all proportions, utility scores, and probabilities; a gamma distribution was used for costs, and resource utilization, and a lognormal distribution was used for relative risk parameters. A tornado diagram was used to represent the sensitivity of individual parameters (OWSA). For PSA, 10,000 Monte Carlo simulations were run and results were depicted using the PSA ICUR plane graph and cost-effectiveness acceptability curve (CEAC). Both net monetary benefit (NMB) (calculated as NMB = Incremental health gain * threshold – incremental costs) and net health benefit (NHB) (calculated as NHB = Incremental health gain – incremental cost/threshold) with 95% CI limits are reported for easier interpretation of cost-effectiveness results.

To assess the feasibility of introduction and financial allocation in the health budget, a budget impact analysis was undertaken at the national level using a societal perspective for a time horizon of five years from 2021 to 2025. A phased uptake of intervention from primary to secondary and then to tertiary level year-on-year was considered. The uptake of intervention was based on PPH numbers proportional to the growth rate of the Indian female population in 15–49 age group over analysis period [51]. The incidence of PPH was assumed to remain constant over this period. This study was reported using the CHEERS 2022 checklist and the model was validated using the AdViSHE tool (see Additional file 2) [52, 53]. As part of the study conduct, the model and findings have been reviewed by members of the HTAIn Technical Appraisal Committee, DHR, MOHFW, India. The study was approved by the institutional ethics board at National Institute for Research in Reproductive and Child Health (NIRRCH). Requisite permissions and approvals from hospitals and state departments for primary data collection were obtained.

## Results

Using HMIS and PPH incidence, it was estimated that an annual cohort of 5,10,915 women would experience primary PPH in Indian public health facilities in the year 2019–20 [15, 54]. For this cohort, a total disaggregated societal cost of INR 3,375,732,060 (USD 48,615,053) would be incurred for overall PPH management with the addition of TXA drug as compared to INR 3,314,015,603 (USD 47,726,254) incurred by the society with standard care treatment, i.e., without TXA intervention. From a health system perspective, this amounts to INR 3,031,950,662 (USD 43,664,142) with the addition of TXA as compared to INR 2,954,109,215 (USD 42,543,121) for the standard care arm. Societal and health system costs incurred per patient between the two decision choices are reported in Table 2. An additional cost of INR 121 (USD 1.74) per patient is incurred with the addition of TXA to PPH management from a disaggregated societal perspective, or INR 152 (USD 2.19) per patient from the health system perspective. For health outcomes, the addition of TXA is associated with undiscounted 44.89 QALYs (discounted 20.25 QALYs) as compared to undiscounted 44.71 QALYs (discounted 20.17 QALYs) associated with standard care. Comparatively, an additional gain of undiscounted 0.18 QALYs per patient (discounted 0.082 QALY) occurs with the addition of TXA to PPH management. Overall, the health system and disaggregated societal costs incurred with the addition of TXA are higher but also associated with a favourable gain in QALYs.

**Table 2:** Costs, consequences, cost-effectiveness, and clinical outcomes for intervention versus standard care treatment for PPH management

	TXA + SOC	SOC
Disaggregated societal perspective		
Total costs of PPH management (for an annual cohort of 510915 PPH cases)	INR 3,375,732,060 (USD 48,615,053)	INR 3,314,015,603 (USD 47,726,254)
Cost of PPH management (per patient)	INR 6607 (USD 95.15)	INR 6486 (USD 93.41)
QALY – undiscounted (per patient)	44.894	44.712
QALY – discounted (per patient)	20.250	20.168
Incremental cost-utility ratio (undiscounted)	INR 663 per QALY gained	
Incremental cost-utility ratio (differential discounting)	INR 1470 per QALY gained	
Health system perspective		
Total costs of PPH management (for an annual cohort of 510915 PPH cases)	INR 3,031,950,662 (USD 43,664,142)	INR 2,954,109,215 (USD 42,543,121)
Cost of PPH management (per patient)	INR 5934 (USD 85.48)	INR 5782 (USD 83.27)
QALY – undiscounted (per patient)	44.894	44.712
QALY – discounted (per patient)	20.250	20.168
Incremental cost-utility ratio (undiscounted)	INR 836 per QALY gained	
Incremental cost-utility ratio (differential discounting)	INR 1854 per QALY gained	
Clinical outcomes		
Total number of surgeries (for an annual cohort of 510915 PPH cases)	19,387 (905 averted)	20,293
Total number of ICU admissions (for an annual cohort of 510915 PPH cases)	27,181 (655 averted)	27,836
Total number of maternal deaths (for an annual cohort of 510915 PPH cases)	13,923 (1990 averted)	15,913

For an incremental societal cost of INR 121 and an incremental discounted gain of 0.082 QALYs, addition of TXA results in an incremental cost per QALY gain, of INR 1,470 per QALY (USD 21.17). This is well below the Indian GDP per capita threshold value (INR 145,742 or USD 2099) suggesting that addition of TXA would be cost-effective in the Indian public health system. Table 2 shows discounted and undiscounted results both from societal and health system perspectives. For clinical outcomes, the model predicts that the addition of TXA for an annual cohort of 510,915 PPH cases would avert 905 surgeries, 655 ICU admissions, and 1990 maternal deaths as compared to without TXA intervention. Table 2 shows the associated clinical outcomes with each decision arm.

**Fig. 2:**
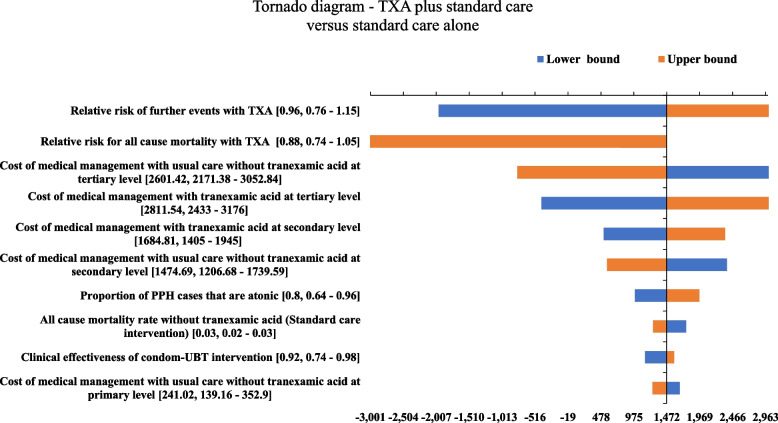
One-way sensitivity analysis for TXA addition versus standard care comparison

**Fig. 3:**
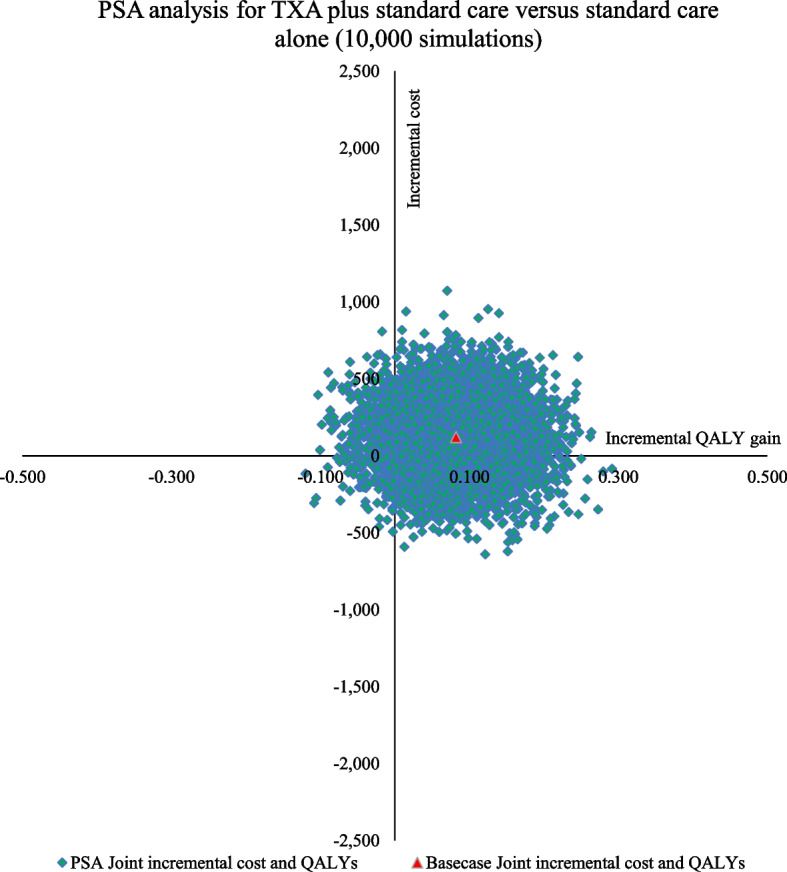
ICUR-QALY plane showing 10,000 Monte Carlo simulations for probabilistic sensitivity analysis

**Fig. 4:**
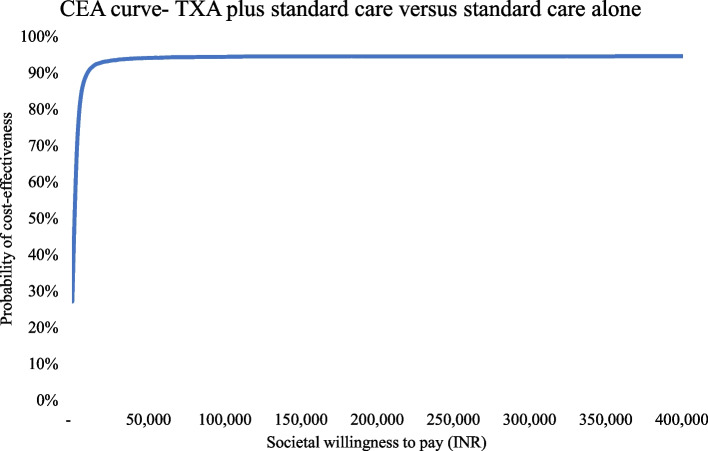
Cost-effectiveness acceptability curve for comparing TXA addition versus standard care PPH treatment

The tornado diagram, shown in Fig. 2, depicts the ten most important parameters affecting cost-effectiveness results. The relative risk of further events with TXA, i.e., control of PPH bleeding without further interventions, the relative risk of all-cause mortality with and without TXA, and the cost of medical management for PPH were the most important parameters affecting cost-effectiveness results. For PSA, an ICUR plane as shown in Fig. 3 shows the results of 10,000 Monte Carlo simulations depicted by green dots with the X axis indicating incremental QALY gain and the Y axis indicating incremental costs incurred. For an Indian willingness to pay (WTP) threshold value of one-time GDP per capita, the analysis suggested that 94.5% of simulations are cost-effective i.e., in favour of the addition of TXA under joint uncertainty, improving the robustness of results. The CEAC graph as shown in Fig. 4 shows cost-effectiveness probability at different societal WTP thresholds. At the current WTP of one-time GDP per capita, there is a 94.6% probability that intervention is cost-effective. This plateaus at 95% for higher thresholds, with a low error probability of 5% around model results. From sensitivity analysis results, NMB and NHB summary statistics with confidence interval limits were calculated. The addition of TXA was found to be associated with a mean NMB value of INR 11,980 or USD 172.5 (95% CI 11,833 to 12,128 or USD 170.4 to 174.7) and a mean NHB value of 0.0822 (95% CI 0.0812 to 0.0832). Positive net benefit framework results indicate that TXA intervention is cost-effective.

Budget impact analysis using phased uptake of intervention in the current mix of PPH management would require a cumulative increase of 2.3% total costs currently incurred for PPH management in India. The absolute budget increase, as shown in Table 3, occurs during the first three years of intervention scale-up. The budget impact then correspondingly stabilizes as per PPH incidence.

**Table 3:** Eligible number of patients, total costs, and budget impact of adding TXA to standard care treatment for PPH management

	2021	2022	2023	2024	2025
Total number of PPH patients in India	643,226	676,439	666,150	596,409	471,710
Current Scenario—patients eligible for available standard care	643,226 (100%)	676,439 (100%)	666,150 (100%)	596,409 (100%)	471,710 (100%)
Bottom-up uptake of TXA intervention	119,704 (19%)	348,637 (52%)	666,150 (100%)	596,409 (100%)	471,710 (100%)
Standard treatment care uptake with TXA introduction	523,521 (81%)	327,803 (48%)	0 (0%)	0 (0%)	0 (0%)
	**The total cost of PPH management with tranexamic acid addition**	**The total cost of PPH management without tranexamic acid addition**	**Budget impact (difference)**	**Percentage impact (% difference)**	
**2021**	INR 4,212,816,600 USD 60,670,189	INR 4,187,661,060 USD 60,307,916	INR 25,155,540 USD 362,273	0.6%	
**2022**	INR 4,477,159,957 USD 64,477,087	INR 4,403,894,864 USD 63,421,972	INR 73,265,093 USD 1,055,115	1.7%	
**2023**	INR 4,476,894,328 USD 64,473,261	INR 4,336,904,772 USD 62,457,225	INR 139,989,556 USD 2,016,037	3.2%	
**2024**	INR 4,008,198,438 USD 57,723,414	INR 3,882,864,695 USD 55,918,441	INR 125,333,742 USD 1,804,973	3.2%	
**2025**	INR 3,170,151,163 USD 45,654,413	INR 3,071,022,610 USD 44,226,830	INR 99,128,552 USD 1,427,584	3.2%	
**Cumulative**	INR 20,345,220,485 USD 292,998,365	INR 19,882,348,001 USD 286,332,383	INR 462,872,485 USD 6,665,982	2.3%	

## Discussion

In light of the landmark WOMAN trial results, WHO updated its clinical guidelines to recommend the addition of intravenous tranexamic acid drug for the medical management of all PPH cases. Given that such an introduction would result in opportunity costs to the health system, the purpose of this study was to estimate costs and consequences for India by conducting a cost-utility analysis to determine whether IV TXA addition for PPH management reflects an efficient allocation of limited available resources.

The analysis found that the introduction of IV TXA for PPH management in Indian public health facilities, with a marginally higher cost and associated improvements in health outcomes, would be cost-effective. These findings were robust across a range of uncertainty analyses undertaken, and it was estimated that the Indian budget allocation for PPH management would incur an additional 2.3 percent expense to provide this intervention. To contextualize the findings of this study, the 2021 systematic review assessing the cost-effectiveness of tranexamic acid in PPH treatment across nine databases identified four studies (two abstracts) from three countries: namely the United States of America (3 studies), Nigeria and Pakistan (1 study) [26]. One of the abstracts identified by this review for the USA setting has also been published since this systematic review [27]. Of these four known studies, the study by Li et al. found TXA to be cost-effective in Nigeria and Pakistan [40]. For the remaining three studies from the USA, Sudhof et al. and Howard et al. reported TXA to be cost-saving whereas the abstract by Wong et al. did not find TXA to be cost-effective [27, 41, 55]. The systematic review reported a cost per QALY value ranging from USD 10.91 per QALY to USD 52,625 per QALY across three studies, all reporting TXA favourable results. The primary outcome measure for this present analysis, i.e., ICUR value of USD 21 per QALY for TXA, is in proximity with the majority of available results especially to 83 per QALY reported for Pakistan and 208 per QALY reported for Nigeria, given their relative comparability to Indian settings as compared to the USA. Clinical event estimates like the number of maternal deaths, surgeries, and ICU admissions reported by this model are relatively higher than expected as compared to those reported by Sudhof et al. and Howard et al. for the USA. Of the limited available evidence, only one study, an abstract by Wong et al. reported TXA to be not cost-effective for the USA, however, the study suggested that TXA may be cost-effective in settings with a higher probability of death due to PPH.

This present study has developed the decision model specifically for the Indian context, taking into account Indian guidelines for cause-specific PPH management by including interventions like local management for traumatic PPH, conservative UBT intervention for atonic PPH, and surgical care as relevant to India, while accounting for expected care delivered based the on level of healthcare facilities accessed by women. The model accounts for expected referrals and ICU admissions based on healthcare facilities accessed by women. Clinical input parameters specific to Indian settings, when available and relevant, have been used in populating the model. The cost input parameters are largely obtained from a primary economic costing study undertaken across healthcare levels in India, thus providing fairly contextual estimates of expected costs. The results are consistent across sensitivity analysis, and the model has been validated for structure, assumptions, and alternate input values.

This cost-effectiveness analysis has its limitations. An important one is regarding the use of clinical effectiveness input parameters for TXA intervention. The WOMAN trial findings did not directly report the clinical effectiveness parameter required to populate the model in terms of control of PPH bleeding without need for any further intervention. This was indirectly calculated from disaggregated data on further interventions after TXA administration as available from trial results and is a sensitive parameter in uncertainty analysis. The WOMAN trial does not specify the inclusion of Indian health facility collaborators in the study, thus suggesting the possibility of clinical result variation for the Indian population. However, as the single largest multi-country RCT from varied settings, this study was found to be the most appropriate source available for this cost-effectiveness analysis. This decision model did not account for any potential adverse events associated with TXA administration, as the WOMAN trial reported no significant differences between the two arms. This inclusion may lead to revised results, however, it is expected that it would not impact it significantly. Saving maternal lives has a significant social and economic impact on families and the larger society. This analysis does not account for all potentially relevant costs and benefits associated with the intervention. The primary costs used in this model are from one context within India and may not be generalizable to all contexts.

Considering that TXA intervention is cost-effective and has been introduced in Indian settings, it is likely to have a positive equity implication. The intervention, given its use in an emergency and its life-saving nature, along with its low cost, is likely to be accepted by all concerned stakeholders. Moreover, there is currently no evidence of any significant adverse effects associated with the TXA drug. The intervention does not require any significant resource inputs in terms of any additional infrastructure, storage, or significant training requirements. The availability of this drug in rural or remote settings where women are at higher risk can benefit significantly. Potential barriers to TXA intervention uptake include a lack of timely drug availability in health facilities, untrained staff providing quality maternal care, and a lack of clear guidelines for its use.

## Conclusion

Early administration of intravenous tranexamic acid to women with primary PPH in Indian public health facilities is recommended from a cost-effectiveness perspective as well as for its potential to avert surgical interventions and save maternal lives. The introduction of IV TXA into the system is expected to require an additional financial investment of 2.3% of the budget that is currently allocated for overall PPH management. Indian policy guidelines, health facility checklists, and training manuals need to be updated, and healthcare personnel need to be sensitized to this recommendation if accepted.

## Supplementary Information

**Additional file 1. **RR parameter calculation for TXA control and RR parameter for death from excel sheet.

**Additional file 2. **Reporting and validation checklists for study.

## Data Availability

The datasets used and/or analysed during the current study are available from the corresponding author on reasonable request.

## Abbreviations

PPH: Postpartum Hemorrhage
WHO: World Health Organization
IV: Intravenous
TXA: Tranexamic Acid
ICUR: Incremental Cost-Utility Ratio
PSA: Probabilistic Sensitivity Analysis
OWSA: One-Way Sensitivity Analysis
SDG: Sustainable Development Goals
MMR: Maternal Mortality Ratio
COVID: Coronavirus Disease
SRS: Sample Registration System
LaQshya: Labor Room Quality Improvement Initiative
JSY: *Janani Suraksha Yojana*
WOMAN Trial: World Maternal Antifibrinolytic Trial
RCOG: Royal College of Obstetricians and Gynecologists
FIGO: International Federation of Gynecology and Obstetrics
MOHFW, India: Ministry Of Health and Family Welfare, India
HTAIn: Health Technology Assessment in India
QALY: Quality Adjusted Life Years
ICU: Intensive Care Unit
PHC: Primary Healthcare Centre
UBT: Uterine Balloon Tamponade
NPPA: National Pharmaceutical Pricing Authority
INR: Indian National Rupees
USD: United States Dollars
GDP: Gross Domestic Product
CI: Confidence Interval
CEAC: Cost-Effectiveness Acceptability Curve
NMB: Net Monetary Benefit
NHB: Net Health Benefit
CHEERS: Consolidated Health Economic Evaluation Reporting Standards
NIRRCH: National Institute for Research in Reproductive and Child Health
WTP: Willingness To Pay
RCT: Randomized Controlled Trial
