# Supplementary material for: Cost-effectiveness and budget impact of adding tranexamic acid for management of post-partum hemorrhage in the Indian public health system
Source: BMC Pregnancy Childbirth. 2023 Jan 6;23:9. doi: 10.1186/s12884-022-05308-4 (PMC9817327; doi:10.1186/s12884-022-05308-4)
Supplement: Supplementary file 1 — Additional file 1. RR parameter calculation for TXA control and RR parameter for death from excel sheet. [file 12884_2022_5308_MOESM1_ESM.docx]

SUPPLEMENTARY MATERIAL – 1

**Input parameter relative risk calculations based on WOMAN Trial study findings (1)**

Table 1: Input parameter calculation for relative risk of further interventions after intravenous Tranexamic acid administration for PPH management (control of PPH bleeding)

| Number of cases | Intervention arm  Tranexamic acid (TXA group) | Placebo group (Standard care) |
| --- | --- | --- |
| Total sample size | 10032 | 9985 |
|  |  |  |
| Uterine balloon tamponade intervention | 705 | 729 |
| Manual removal of placenta | 918 | 961 |
| Embolization | 10 | 13 |
| Brace sutures | 300 | 250 |
| Arterial ligation | 225 | 254 |
| Laparotomy | 82 | 127 |
| Total | 2240 | 2334 |
|  | | |
| Proportion of TXA group requiring further intervention | =2240/10032  =0.223285 | |
| Proportion of SOC group requiring further intervention | =2334/9888  =0.23375 | |
| Relative risk for further intervention with TXA | = 0.955229 | |
| Relative risk for further intervention with SOC | =1.05 | |

Table 2: Input parameter calculation for relative risk of death due to bleeding with intravenous Tranexamic acid administration for PPH management

| Number of cases | Intervention arm Tranexamic acid (TXA group) | | Placebo group (Standard care) |
| --- | --- | --- | --- |
| Total number of cases with intervention provided in less than 1 hour | 4846 | | 4726 |
| Total number of deaths among intervention provided in less than 1 hour | 49 | | 60 |
| Total number of cases with intervention provided in 1-3 hours | 2674 | | 2682 |
| Total number of deaths among intervention provided in 1-3 hours | 40 | | 67 |
| Total number of cases with intervention provided up to 3 hours | 7520 | | 7408 |
| Total number of deaths among intervention provided up to 3 hours | 89 | | 127 |
| Proportion of deaths among TXA group | = 89/7520  = 0.011835 | | |
| Proportion of deaths among SOC group | = 127/7408  = 0.017144 | | |
| Relative risk of maternal deaths up to 3 hours (due to TXA) | = 0.69035 | | |
|  | | | |
| Total sample size | 10036 | 9985 | |
| Total number of deaths at any time (Includes deaths due to bleeding) | 227 | 256 | |
| Proportion of all causes of death among TXA group | = 227/10036  = 0.022618 | | |
| Proportion of all causes of death among SOC group | = 256/9985  =0.025638 | | |
| Relative risk of death with TXA due to all causes | = 0.882212 | | |

**Reference:**

1. Shakur H, Roberts I, Fawole B, Chaudhri R, El-Sheikh M, Akintan A, et al. Effect of early tranexamic acid administration on mortality, hysterectomy, and other morbidities in women with post-partum haemorrhage (WOMAN): an international, randomised, double-blind, placebo-controlled trial. The Lancet [Internet]. 2017 May 27 [cited 2022 Apr 15];389(10084):2105–16. Available from: http://www.thelancet.com/article/S0140673617306384/fulltext
