# Supplementary material for: Cost-effectiveness and budget impact of adding tranexamic acid for management of post-partum hemorrhage in the Indian public health system
Source: BMC Pregnancy Childbirth. 2023 Jan 6;23:9. doi: 10.1186/s12884-022-05308-4 (PMC9817327; doi:10.1186/s12884-022-05308-4)
Supplement: Supplementary file 2 — Additional file 2. Reporting and validation checklists for study. [file 12884_2022_5308_MOESM2_ESM.doc]

**CHECKLISTS FOR STUDY VALIDATION AND REPORTING**

**Part-1**

**CHEERS STATEMENT**

CHEERS checklist—Items to include when reporting economic evaluations of health interventions

| **Section/item** | **Item No** | **Guidance for Reporting** | **Reported on page No/ line No** |
| --- | --- | --- | --- |
| **TITLE** | | | |
| Title | 1 | Identify the study as an economic evaluation and specify the intervention being compared. | Title  Page no. 01, Line no. 01 to 02 |
| **ABSTRACT** | | | |
| Abstract | 2 | Provide a structured summary that highlights context, key methods, results and alternative analyses | Abstract  Page no. 03 & 04, Line no. 54 to 85 |
| **INTRODUCTION** | | | |
| Background and objectives | 3 | Give the context for the study, the study question and its practical relevance for decision making in policy or practice | Background  Page no. 06, Line no. 138 to 150  Page no. 07, Line no. 151 to 155 |
| **METHODS** | | | |
| Health Economic Analysis Plan | 4 | Indicate whether a health economic analysis plan was developed and where available | HEAP was not developed a priori. |
| Study Population | 5 | Describe characteristics of the study population (such as age range, demographics, socioeconomic or clinical characteristics) | Methods  Page no. 07, Line no. 163 to 165 |
| Setting and location | 6 | Provide relevant contextual information that may influence findings | Methods  Page no. 07, Line no. 163 to 165  Page no. 08, Line no. 188 to 192 |
| Comparators | 7 | Describe the interventions or strategies being compared and why they were chosen. | Methods  Page no. 07, Line no. 158 to 160  Page no. 07, Line no. 169 to 175 |
| Perspective | 8 | State the perspective(s) adopted by the study and why chosen | Methods  Page no. 07, Line no. 161 to 163 |
| Time horizon | 9 | State the time horizon for the study and why appropriate. | Methods  Page no. 08, Line no. 179 to 181 |
| Discount rate | 10 | Report the discount rate(s) and reason chosen | Methods  Page no. 11, Line no. 261 to 266 |
| Selection of outcomes | 11 | Describe what outcomes were used as the measure(s) of benefit(s) and harm(s) | Methods  Page no. 07, Line no. 175  Page no. 08, Line no. 176 to 179 |
| Measurement of outcomes | 12 | Describe how outcomes used to capture benefit(s) and harm(s) were measured | Methods  Page no. 10, Line no. 248 to 249  Page no. 11, Line no. 250 to 255 |
| Valuation of outcomes | 13 | Describe the population and methods used to measure and value outcomes. | Methods  Page no. 11, Line no. 256 to 261 |
| Measurement and valuation of resources and costs | 14 | Describe how costs were valued | Methods  Page no. 10, Line no. 228 to 246 |
| Currency, price date, and conversion | 15 | Report the dates of the estimated resource quantities and unit costs, plus the currency and year of conversion. | Methods  Page no. 10, Line no. 235 to 239  Page to 11, Line no.258  Page to 11, Line no 268 to 269 |
| Rationale and Description of model | 16 | If modelling is used, describe in detail and why used. Report if the model is publicly available and where it can be accessed | Background  Page no. 7, Line no. 144 to 148  Methods  Page no.7, Line no.158 to 161  Page no. 08, Line no. 187 to 200  Page no. 09, Line no. 201 to 206  Declaration  Page no 20, Line no. 474 to 475  Figure 1 |
| Analytics and Assumptions | 17 | Describe any methods of analysing or statistically transforming data, any extrapolation methods and approaches for validating any model used | Methods  Page no.11, Line no.256 to 269  Page no.12, Line no. 290 to 292 |
| Characterizing Heterogeneity | 18 | Describe any methods used for estimating how the results of the study vary for sub-groups | Methods  Page no. 07, Line no. 165 to 169  The study does not assess the other subgroup based on time of TXA administration for lack of all relevant input parameters |
| Characterizing distributional effects | 19 | Describe how impacts are distributed across different individuals or adjustments made to reflect priority populations | Time of TXA administration has an important outcome impact. Only effect of early administration for available input parameters has been assessed. |
| Characterizing uncertainty | 20 | Describe methods to characterize any sources of uncertainty in the analysis | Methods  Page no. 11, Line no. 270 to 274  Page no. 12, Line no. 275 to 283 |
| Approach to engagement with patients and others affected by the study | 21 | Describe any approaches to engage patients or service recipients, the general public, communities, or stakeholders (e.g., clinicians or payers) in the design of the study. | Methods  Page no. 12, Line no. 291 to 292 |
| **RESULTS** | | | |
| Study parameters | 22 | Report all analytic inputs (e.g., values, ranges, references) including uncertainty or distributional assumptions | Table 1 |
| Summary of main results | 23 | Report the mean values for the main categories of costs and outcomes of interest and summarise them in the most appropriate overall measure. | Results  Page no. 12, Line no. 298 to 299  Page no. 13, Line no. 300 to 323  Table 2  Table 3 |
| Effect of uncertainty | 24 | Describe how uncertainty about analytic judgments, inputs, or projections affect findings. Report the effect of choice of discount rate and time horizon, if applicable | Results  Page no. 14, Line no. 324 to 341  Figure 2  Figure 3  Figure 4 |
| Effect of engagement with patients and others affected by the study | 25 | Report on any difference patient/service recipient, general public, community, or stakeholder involvement made to the approach or findings of the study | Not undertaken |
| **DISCUSSION** | | | |
| Study findings, limitations, generalisability, and current knowledge | 26 | Report key findings, limitations, ethical or equity considerations not captured, and how these could impact patients, policy, or practice. | Discussion  Page no. 15, Line no. 350 to 373,  Page no. 16, Line no. 374 to 398  Page no. 17, Line no. 399 to 416 |
| **OTHER RELEVANT INFORMATION** | | | |
| Source of funding | 27 | Describe how the study was funded and any role of the funder in the identification, design, conduct, and reporting of the analysis. | Declaration  Page no. 21, Line no. 481 to 485 |
| Conflicts of interest | 28 | Report authors conflicts of interest according to journal or International Committee of Medical Journal Editors requirements. | Declaration  Page no.21, Line no. 478 |

**Part-2**

**AdViSHE TOOL**

**Assessment of the Validation Status of Health-Economic decision models**

AdViSHE is a questionnaire that modellers can complete to report on the efforts performed to improve the validation status of their health-economic (HE) decision model. It is not intended to replace validation by model users but rather to inform the direction of validation efforts and to provide a baseline for replication of the results. In addition to using it after a model is finished, the modellers can use AdViSHE to guide validation efforts during the modelling process.

The modellers are asked to comment on the validation efforts performed while building the underlying HE decision model and afterwards. Many of the questions simply refer to the model documentation. AdViSHE is divided into five parts, each covering an aspect of validation:

- Part A: Validation of the conceptual model (2 questions)

- Part B: Input data validation (2 questions)

- Part C: Validation of the computerized model (4 questions)

- Part D: Operational validation (4 questions)

- Part E: Other validation techniques (1 question)

No final validation score is calculated, as the assessment of the answers and the overall validation effort is left to the model users. It is assumed that the model has been built according to prevailing modelling and reporting guidelines. Some questions may not be applicable to a particular model. If this is the case, the model builder should take the opt-out option and provide a justification of why this item is not deemed applicable.

**Table:** Author responses to the Advishe Model validation tool

|  | **AdViSHE tool** | **Model validation for intravenous tranexamic acid use in management of PPH in India** |
| --- | --- | --- |
|  | Part A: Validation of the conceptual model (2 questions)  Part A discusses techniques for validating the conceptual model. A conceptual model describes the underlying system (e.g., progression of disease) using a mathematical, logical, verbal, or graphical representation. Please indicate where the conceptual model and its underlying assumptions are described and justified. | The conceptual model and schematic diagram of the decision analytic model is described in the manuscript along with the figure of the model (Figure 1) |
| A1 | Face validity testing (conceptual model): Have experts been asked to judge the appropriateness of the conceptual model?  If yes, please provide information on the following aspects:  - Who are these experts?  - What is your justification for considering them experts?  - To what extent do they agree that the conceptual model is appropriate?  If no, please indicate why not.  Aspects to judge include: appropriateness to represent the underlying clinical process/disease (disease stages, physiological processes, etc.); and appropriateness for economic evaluation (comparators, perspective, costs covered, etc.). | Yes.  The HTAIn (Health Technology Assessment of India) division of the Department of Health Research of the Ministry of Health and Family Welfare, Govt of India constituted the Technical Appraisal Committee to critically assess the model, assumptions as well the whole economic evaluation to provide evidence to the government user Department for updating the policy guidelines available.  The experts constitute a mix of clinicians, health economists, policy makers from the Indian context who are experienced & well familiarized with the Indian settings. They constitute the committee which critically examines and recommends the topics to the highest decision-making authority which recommends the same for implementation in India.  Fully agree  Not applicable |
| A2 | Cross validity testing (conceptual model): Has this model been compared to other conceptual models found in the literature or clinical textbooks?  If yes, please indicate where this comparison is reported.  If no, please indicate why not. | The current model can be compared to other conceptual models reported in the literature however is specific to the Indian contextual settings.  The comparison to existing literature is described in discussion section of the manuscript. |
|  | Part B: Input data validation (2 questions)  Part B discusses techniques to validate the data serving as input in the model. These techniques are applicable to all types of models commonly used in Health economic modelling.  Please indicate where the description and justification of the following aspects are given:  - search strategy;  - data sources, including descriptive statistics;  - reasons for inclusion of these data sources;  - reasons for exclusion of other available data sources;  - assumptions that have been made to assign values to parameters for which no data was available;  - distributions and parameters to represent uncertainty;  - data adjustments: mathematical transformations (e.g., logarithms, squares); treatment of outliers; treatment of missing data; data synthesis (indirect treatment comparison, network meta-analysis); calibration; etc. | Details regarding search strategy; data sources, including descriptive statistics; distributions and parameters to represent uncertainty, assumptions that have been made to assign values to parameters for which no data was available, reasons for inclusion or exclusion of data sources is presented in the methods section of the manuscript and also mentioned in limitations.  Treatment of outliers; treatment of missing data; data synthesis (indirect treatment comparison, network meta-analysis); calibration were not applicable to analysis of this sudy.  Available in the excel sheet and will be provided upon reasonable request |
| B1 | Face validity testing (input data): Have experts been asked to judge the appropriateness of the input data?  If yes, please provide information on the following aspects:  - Who are these experts?  - What is your justification for considering them experts?  - To what extent do they agree that appropriate data has been used?  If no, please indicate why not.  Aspects to judge may include but are not limited to: potential for bias; generalizability to the target population; availability of alternative data sources; any adjustments made to the data. | The HTAIn (Health Technology Assessment of India) division of the Department of Health Research of the Ministry of Health and Family Welfare, Govt of India constituted the Technical Appraisal Committee to critically assess the model, assumptions as well the whole economic evaluation to provide evidence to the government user department for updating the policy guidelines available.  The experts constitute a mix of clinicians , health economists, policy makers from the Indian context who are experienced & well familiarized with the Indian settings. They constitute the committee which critically examines and recommends the topics to the highest decision making authority which recommends the same for implementation in India.  Fully Agree  Not Applicable |
| B2 | Model fit testing: When input parameters are based on regression models, have statistical tests been performed?  If yes, please indicate where the description, the justification and the outcomes of these tests are reported.  If no, please indicate why not  Examples of regression models include but are not limited to: disease progression based on survival curves; risk profiles using regression analysis on a cohort; local cost estimates based on multi-level models; meta-regression; quality-of-life weights estimated using discrete choice analysis; mapping of disease-specific quality-of-life weights to utility values.  Examples of tests include but are not limited to: comparing model fit parameters (R2, AIC, BIC); comparing alternative model specifications (covariates, distributional assumptions); comparing alternative distributions for survival curves (Weibull, lognormal, logit); testing the numerical stability of the outcomes (sufficient number of iterations); testing the convergence of the regression model; visually testing model fit and/or regression residuals. | Input parameters (except for cost) are taken/calculated from data available in literature as cited in the methods section. Cost parameters are calculated using data collected for a primary study done by the authors published here: <https://bmjopen.bmj.com/content/bmjopen/11/3/e042389.full.pdf>  These methods are described in the methods section of the manuscript. |
|  | Part C: Validation of the computerized model (4 questions)  Part C discusses various techniques for validating the model as it is implemented in a software program. If there are any differences between the conceptual model (Part A) and the final computerized model, please indicate where these differences are reported and justified. | There is no difference observed between the conceptualized and computerized versions |
| C1 | External review: Has the computerized model been examined by modelling experts?  If yes, please provide information on the following aspects:  - Who are these experts?  - What is your justification for considering them experts?  - Can these experts be qualified as independent?  - Please indicate where the results of this review are reported, including a discussion of any unresolved issues.  If no, please indicate why not.  Aspects to judge may include but are not limited to: absence of apparent bugs; logical code structure optimized for speed and accuracy; appropriate translation of the conceptual model. | Yes.  The computerised model was examined by independent health economists who are experienced in health economic modeling  They are experts in health economics who are familiarized and have experience of working in the Indian settings  Yes  It is reported in the discussion & scknowledgements section and there are no unresolved issues |
| C2 | Extreme value testing: Has the model been run for specific, extreme sets of parameter values in order to detect any coding errors?  If yes, please indicate where these tests and their outcomes are reported.  If no, please indicate why not.  Examples include but are not limited to: zero and extremely high (background) mortality; extremely beneficial, extremely detrimental, or no treatment effect; zero or extremely high treatment or healthcare costs. | Written in methodology /results  The excel sheet can be provided based on reasonable request |
| C3 | Testing of traces: Have patients been tracked through the model to determine whether its logic is correct?  If yes, please indicate where these tests and their outcomes are reported.  If no, please indicate why not.  In cohort models, this would involve listing the number of patients in each disease stage at one, several, or all time points (e.g., Markov traces). In individual patient simulation models, this would involve following several patients throughout their natural disease progression. | Yes, women have been tracked through the Microsoft Excel model and logic was been found to be correct.  The model described is a decision tree model and we are looking at a one time event - PPH post pregnancy. |
| C4 | Unit testing: Have individual sub-modules of the computerized model been tested?  If yes, please provide information on the following aspects:  - Was a protocol that describes the tests, criteria, and acceptance norms defined beforehand?  - Please indicate where these tests and their outcomes are reported.  If no, please indicate why not.  Examples include but are not limited to: turning sub-modules of the program on and off; altering global parameters; testing messages (e.g., warning against illegal or illogical inputs), drop-down menus, named areas, switches, labelling, formulas and macros; removing redundant elements. | Yes a protocol that describes the tests, criteria, and acceptance norms was defined beforehand  The tests, criteria, acceptance norms &outcomes are reported in methodology & results section. |
|  | Part D: Operational validation (4 questions)  Part D discusses techniques used to validate the model outcomes. |  |
| D1 | Face validity testing (model outcomes): Have experts been asked to judge the appropriateness of the model outcomes?  If yes, please provide information on the following aspects:  - Who are these experts?  - What is your justification for considering them experts?  - To what extent did they conclude that the model outcomes are reasonable?  If no, please indicate why not.  Outcomes may include but are not limited to: (quality-adjusted) life years; deaths; hospitalizations; total costs. | Yes.  The HTAIn (Health Technology Assessment of India) division of the Department of Health Research of the Ministry of Health and Family Welfare, Govt of India constituted the Technical Appraisal Committee to critically assess the model, assumptions as well the whole economic evaluation to provide evidence to the government user department for updating the policy guidelines available.  The experts constitute a mix of clinicians, health economists, policy makers from the Indian context who are experienced & well familiarized with the Indian settings. They constitute the committee which critically examines and recommends the topics |
| D2 | Cross validation testing (model outcomes): Have the model outcomes been compared to the outcomes of other models that address similar problems?  If yes, please provide information on the following aspects:  - Are these comparisons based on published outcomes only, or did you have access to the alternative model?  - Can the differences in outcomes between your model and other models be explained?  - Please indicate where this comparison is reported, including a discussion of the comparability with your model.  If no, please indicate why not.  Other models may include models that describe the same disease, the same intervention, and/or the same population. | Yes. Reported in the discussion  The comparisons are based on the published outcomes in light of the Indian settings.  Yes  Reported in Discussion |
| D3 | Validation against outcomes using alternative input data: Have the model outcomes been compared to the outcomes obtained when using alternative input data?  If yes, please indicate where these tests and their outcomes are reported.  If no, please indicate why not.  Alternative input data can be obtained by using different literature sources or datasets, but can also be constructed by splitting the original data set in two parts, and using one part to calculate the model outcomes and the other part to validate against. | Yes. Reported in Methods, Results & Discussion |
| D4 | Validation against empirical data: Have the model outcomes been compared to empirical data?  If yes, please provide information on the following aspects:  - Are these comparisons based on summary statistics, or patient-level datasets?  - Have you been able to explain any difference between the model outcomes and empirical data?  - Please indicate where this comparison is reported.  If no, please indicate why not. | No, the data has been not validated against empirical data. However, findings have been compared against similar analysis undertaken across literature and these are reported in the discussion section of the manuscript. |
| D4A | Comparison against the data sources on which the model is based (dependent validation). | Sensitivity analysis (OWSA/PSA) is done for input parameters based on confidence intervals reported /derived from literature.  Findings have been compared against similar analysis undertaken across literature that use similar data inputs and this is reported in discussion section of the manuscript. |
| D4B | Comparison against a data source that was not used to build the model (independent validation). | A sensitivity analysis (OWSA/PSA)is done for input parameters based on confidence intervals reported /derived from literature. |
|  | Part E: Other validation techniques (1 question) |  |
| E1 | Other validation techniques: Have any other validation techniques been performed?  If yes, indicate where the application and outcomes are reported, or else provide a short summary here.  Examples of other validation techniques: structured “walk-throughs” (guiding others through the conceptual model or computerized program step-by-step); naïve benchmarking (“back-of-the-envelope” calculations); heterogeneity tests; double programming (two model developers program components independently and/or the model is programmed in two different software packages to determine if the same results are obtained). | Structured “walk-throughs” (guiding others through the conceptual model or computerized program step-by-step) was presented to trained technical resource hub personnel undertaking health economic evaluations before presenting it separately before the Technical Appraisal Committee  Additional validation using extreme values to assess model structure and calculations was undertaken. Validation using the Cheers Checklist 2022 for reporting was also undertaken.  . |
